# Gene Expression Profiling Reveals Epithelial Mesenchymal Transition (EMT) Genes Can Selectively Differentiate Eribulin Sensitive Breast Cancer Cells

**DOI:** 10.1371/journal.pone.0106131

**Published:** 2014-08-29

**Authors:** Zoltán Dezső, Judith Oestreicher, Amy Weaver, Stephanie Santiago, Sergei Agoulnik, Jesse Chow, Yoshiya Oda, Yasuhiro Funahashi

**Affiliations:** Biomarkers and Personalized Medicine Core Function Unit, Eisai Product Creation Systems, Eisai Inc., Andover, Massachusetts, United States of America; University of Nebraska Medical Center, United States of America

## Abstract

**Objectives:**

Eribulin mesylate is a synthetic macrocyclic ketone analog of the marine sponge natural product halichondrin B. Eribulin is a mechanistically unique inhibitor of microtubule dynamics. In this study, we investigated whether selective signal pathways were associated with eribulin activity compared to paclitaxel, which stabilizes microtubules, based on gene expression profiling of cell line panels of breast, endometrial, and ovarian cancer in vitro.

**Results:**

We determined the sets of genes that were differentially altered between eribulin and paclitaxel treatment in breast, endometrial, and ovarian cancer cell line panels. Our unsupervised clustering analyses revealed that expression profiles of gene sets altered with treatments were correlated with the in vitro antiproliferative activities of the drugs. Several tubulin isotypes had significantly lower expression in cell lines treated with eribulin compared to paclitaxel. Pathway enrichment analyses of gene sets revealed that the common pathways altered between treatments in the 3 cancer panels were related to cytoskeleton remodeling and cell cycle regulation. The epithelial-mesenchymal transition (EMT) pathway was enriched in genes with significantly altered expression between the two drugs for breast and endometrial cancers, but not for ovarian cancer. Expression of genes from the EMT pathway correlated with eribulin sensitivity in breast cancer and with paclitaxel sensitivity in endometrial cancer. Alteration of expression profiles of EMT genes between sensitive and resistant cell lines allowed us to predict drug sensitivity for breast and endometrial cancers.

**Conclusion:**

Gene expression analysis showed that gene sets that were altered between eribulin and paclitaxel correlated with drug in vitro antiproliferative activities in breast and endometrial cancer cell line panels. Among the panels, breast cancer provided the strongest differentiation between eribulin and paclitaxel sensitivities based on gene expression. In addition, EMT genes were predictive of eribulin sensitivity in the breast and endometrial cancer panels.

## Introduction

Eribulin mesylate (eribulin) is a synthetic macrocyclic ketone analog of the marine sponge natural product halichondrin B and an inhibitor of microtubule dynamics [1], [2]. Eribulin inhibits microtubule dynamics through a novel mechanism relative to other tubulin-targeting agents, including the taxanes and vinca alkaloids, by specifically binding with high affinity to the plus ends of microtubules and thereby suppressing microtubule dynamics and leading to inhibition of microtubule growth in the absence of effects on microtubule shortening at microtubule plus ends, and formation of nonproductive tubulin aggregates [3], [2]. This results in G2-M cell-cycle arrest, disruption of normal mitotic spindles, and induction of apoptosis. Eribulin (as Halaven) has been approved in a number of countries worldwide for the treatment of certain patients with advanced breast cancer. In breast cancer, the use of taxanes and anthracyclines is often effective early on, although resistance to these agents commonly limits their potential at late-line settings [4], and as a result the prognosis for metastatic breast cancer remains poor. Knowledge of eribulin’s unique interactions with microtubules compared to other tubulin-binding drugs has generated interest in the possibility that eribulin may have a unique spectrum of anticancer activities and may provide new treatment options for patients who are resistant to other tubulin binding agents. In this study we have focused on comparing eribulin to paclitaxel, a taxane widely used for breast cancer and gynecological cancers such as ovarian and endometrial cancers, which stabilizes microtubule polymers leading to mitotic arrest and apoptosis [5], [6]. Several studies have identified gene mutations and expression patterns associated with paclitaxel resistance, with some of the biomarkers predictive of such resistance including beta-tubulin mutations [7]–[9], overexpression of beta-3-tubulin [10], overexpression of the microtubule-associated protein stathmin [11], upregulation of ErbB2 [12], and mutation of p53 [13], [14]. Although numerous studies have focused on paclitaxel resistance, molecular biomarkers for predicting efficacy of eribulin, or the relative benefit of eribulin compared with paclitaxel, have not yet been reported.

In this study our goal was to investigate whether selective pathways were associated with eribulin activity compared to paclitaxel and to find potential biomarkers predictive of eribulin sensitivity or resistance when compared to paclitaxel. We performed gene expression profiling of breast, ovarian, and endometrial cancer cell line panels treated with eribulin and paclitaxel. In addition, we measured in vitro anti proliferative activities (IC_50_) for both eribulin and paclitaxel for the same cell line panels. High throughput expression profiles combined with in vitro proliferation data represents an unbiased approach that enables us to identify multiple components of pathways with altered expression that may be selectively activated by eribulin compared to paclitaxel and that may contribute to cellular susceptibility to the drugs. Furthermore, the inclusion of 3 cancer panels in our analyses allowed us to compare the altered pathway profiles identified for each cancer type. Our first approach was to identify genes with significantly changed expression between the two compounds for each cancer panel and perform functional pathway analyses. Next, gene sets and affected signaling pathways were correlated with in vitro antiproliferative data to investigate if any of the differentially affected pathways were associated with drug resistance. Finally, we confirmed our main findings in the breast cancer panel by qPCR.

## Methods

### In vitro antiproliferative activity

In vitro antiproliferative activities (IC_50_) were determined in 27 breast, 19 endometrial, and 21 ovarian cancer cell lines treated with eribulin and paclitaxel. Three hours after plating, serial dilutions of tested compounds were added. Each experiment was performed in triplicate. Mean IC_50_ values and 95% confidence intervals (CI) were calculated based on IC_50_ values generated from separate sigmoidal curves representing the growth inhibition activity versus eribulin and paclitaxel concentrations in 3 independent experiments. Statistical analyses were performed using GraphPad Prism version 5.02 (GraphPad Software, San Diego, CA). IC_50_ values for all 3 cancer panels are included in Table S1.

### Gene expression profiling (Affymetrix)

We performed gene expression analysis of 27 breast, 19 endometrial, and 21 ovarian cancer cell lines treated with eribulin or paclitaxel for 24 hours at concentrations of 10×IC_50_. Total RNA was extracted using RNeasy Mini kit (Qiagen) and was used to prepare biotinylated fragmented cRNA for analysis on Human Genome U133 plus 2.0 arrays (Affymetrix). Three technical replicates were included for eribulin, paclitaxel, and untreated cell lines. Arrays were processed according to the GeneChip Expression Analysis Technical Manual (Affymetrix). Each gene chip was analyzed using Affymetrix Microarray Analysis Suite version 5.0 to obtain raw data. Genes with average expression <100 were considered not expressed and excluded from the analysis. Gene expression data from these studies are available at the GEO public repository website (accession number GSE50832).

### Quantitative (q)PCR analysis

We extracted genes from the “Development Regulation of epithelial-to-mesenchymal transition (EMT)” pathway from the knowledge base of the commercially available Metacore. There were 36 out of 92 genes expressed in breast cancer cell lines based on the Affymetrix array (intensity >100). Effects of eribulin and paclitaxel on the expression profiles of these 36 genes from the EMT pathways and 8 tubulin isotypes was confirmed in the breast cancer panel by qPCR using Custom TaqMan Low Density Cards (TLDA) with ABI7900HT (Life Technologies) instruments. The 27 breast cancer cell lines were treated with eribulin and paclitaxel for 24 hours at concentrations of 10×IC_50_. Three technical replicates were included for eribulin, paclitaxel, and untreated cell lines. We calculated ΔCT values for sample-to-sample normalization based on the average CT values of 4 endogenous controls (GAPDH, GUSB, HPRT1, PPIA). Statistical analysis and clustering were performed on ΔCT values. Fold changes were calculated after 2^−ΔCT^ transformation into linear space. The ΔCT values for the 2 treatments in the 3 cancer panels are included in Table S2. The clustering result of the EMT pathway was based on the spearman distance metric.

To investigate long-term effects of compounds on expression of EMT-related genes, five breast cancer cells (HCC1806, BT474, HCC38, HCC70 and AU565) were incubated in the presence of eribulin at 1×IC_50_ for 7 days. All treatments were done in triplicates. Total RNA was isolated using the RNeasy Mini kit (Qiagen, Valencia, CA, USA). Reverse transcription was carried out using the High Capacity cDNA Reverse Transcription kit (Life Technologies, Grand Island, NY, USA). Gene expression was measured with Taqman probes on Custom TaqMan Low Density Cards (TLDA) using ABI7900 (Applied Biosystems, Life Technologies). We calculated ΔCT values for sample-to-sample normalization based on the CT values of ACTB endogenous control (Table S4).

### Statistical and pathway analysis of gene expression

Gene expressions were analyzed using Expressionist software (Genedata) and R statistical software [15]. After data normalization log2 ratios were calculated for both treatments normalized to control. To identify genes significantly altered between treatments we applied paired t-tests between the log transformed expression ratios of the treated cell lines for each cancer cell line panel separately. Genes with fold change (FC) >1.5 between treatments for at least one cell line and p value <0.01 based on the paired t-test were considered significantly altered between treatments (Table S3). Pathway analysis of significantly altered genes was performed using Metacore commercial software. Pathways were considered significant based on p<0.05 false discovery rate (FDR) adjusted threshold for multiple comparison correction. Cell lines were clustered based on their gene expression profiles by unsupervised hierarchical clustering, where the distance metric was the Pearson distance and both the “ward” and “average” linkage criteria was used. We picked in each case the algorithm which gave the highest average silhouette (using the “cluster” library in R), a measure characterizing how tightly the data are grouped in the clusters. In order to be able to perform statistical tests between the drug sensitivity of the cell line clusters we considered only results where the clusters contained at least three cell lines with associated IC_50_ data. The best clustering was retained and the top two and three clusters in the hierarchical tree were correlated with in vitro antiproliferative activity. A t-test was applied to identify significant differences between the cell line clusters.

### Predictions of drug sensitivity based on signature genes

We tested the predictive power of the EMT pathway genes based on the elastic net regression method [18]. This method is suited for cases where the number of features (genes) is greater than the number of samples and some of the features are highly correlated. The algorithm combines two penalty terms to strike a balance between the need to generate a sparse model, but at the same time retaining groups of correlated genes. We used this method because the number of genes was larger than the number of samples, and because the genes were extracted from the same pathway we expected a high correlation among the expression profiles. We used the “glmnet” package [16] in R, where the regression model is built based on two parameters (α, λ). The first parameter (α) controls the relative strength of the two penalty terms (α = 0 and 1 corresponds to the ridge and lasso regression, respectively). The second parameter (λ) controls the overall strength of the penalties [17]. We performed a leave-one-out cross validation to evaluate the prediction power of the gene sets. To predict the sensitivity of the left out sample we generated a sequence of 100 values of α between 0 and 1 and for each α value a λ was chosen to minimize the mean square error based on a 10 fold cross-validation of the training data (total number of sample minus one). For this step, 100 values of λ were tested based on the sequence generated by the default setting in the “glmnet” package. The α and λ pair which had the smallest mean square error was retained for prediction. The performance of each model was evaluated using the Pearson correlation coefficient between the predicted values and the measured IC_50_ values.

## Results

### Comparison of gene signatures and correlation with drug sensitivity

**Figure 1:**
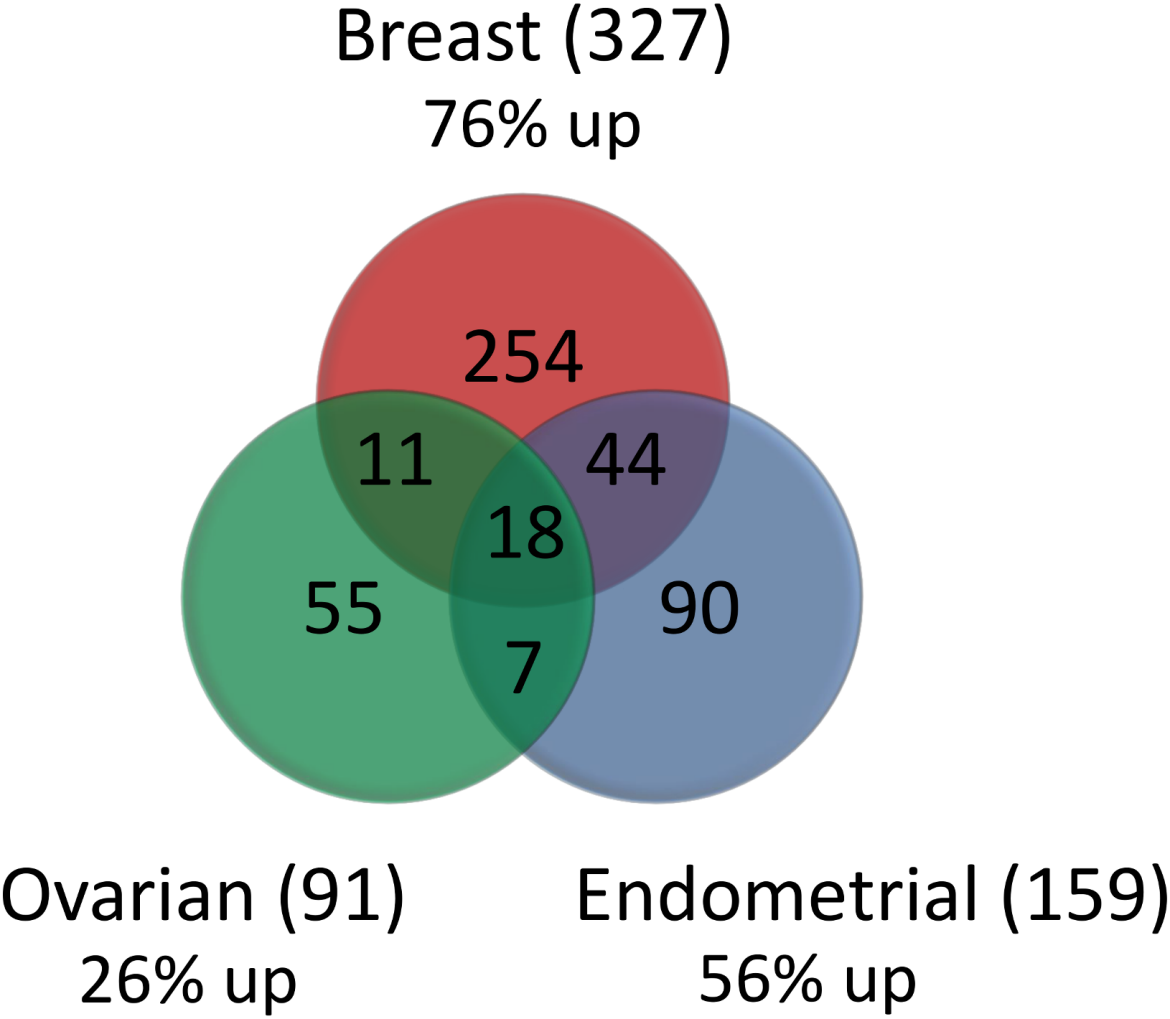
Overlap among gene signatures for the 3 cancer panels. We identified sets of genes with significantly altered gene expression profiles between eribulin and paclitaxel treatments for breast, ovarian, and endometrial cancer. The signature consisted of 327, 91, and 159 genes for breast, ovarian, and endometrial cancer, respectively. The percentage of genes having higher expression in cell lines treated with eribulin compared to paclitaxel is 76%, 56%, and 26% for breast, endometrial, and ovarian cancer, respectively.

**Figure 2:**
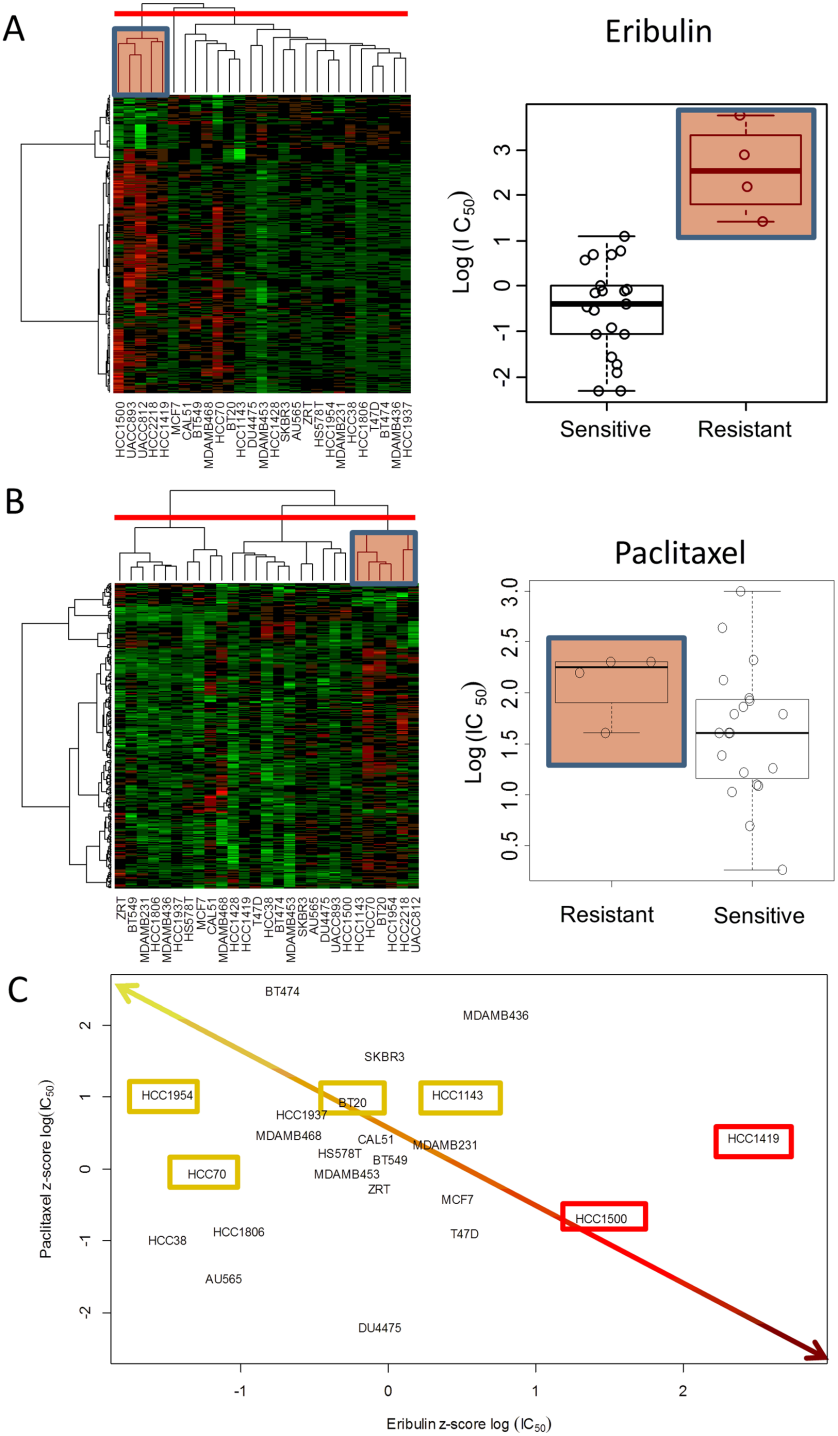
Correlation of expression profiles with drug sensitivity. A) Correlation of eribulin signature with drug sensitivity. Hierarchical clustering of eribulin expression signature identified two clusters of cell lines with significantly different sensitivity to eribulin (p = 0.004). The red box indicates the eribulin resistant cluster. B) Correlation of paclitaxel signature with drug sensitivity. Hierarchical clustering of paclitaxel expression signature identified cluster with differences of paclitaxel sensitivity (p = 0.06). The red box indicates the most paclitaxel resistant cell line cluster. C) Scatter plot of eribulin and paclitaxel sensitivity. Cell lines located in the upper left corner are the most resistant to paclitaxel as compared to eribulin, and cell lines located at the lower right corner are the most resistant to eribulin as compared to paclitaxel. Yellow and red boxes indicate the cell lines identified based on expression profiles as uniquely resistant to paclitaxel and eribulin, respectively.

Our goal was to identify selective signaling pathways associated with eribulin compared to paclitaxel in the 3 cell line panels based on the gene expression profiling. We started the analysis by identifying the genes differentially altered between treatments with eribulin and paclitaxel. We performed a paired t-test for each cancer panel and calculated fold changes for each cell line separately between the two treatments. After applying a threshold on p values and fold-changes (p<0.01 and FC>1.5), we defined the gene signatures consisting of 91, 159, and 327 genes for ovarian, endometrial, and breast cancer cell lines, respectively (Figure 1, Table S3). A large fraction of the genes were unique: 78% of genes from signatures were unique to breast, 57% to endometrial and 60% to ovarian cancer. There was only a small overlap (18 genes). The gene signatures also showed differences in the direction of alteration between the drugs in the three cancer panel. We compared the number of genes up- or down-regulated in at least one cell line (FC>1.5) between treatments and found that in breast and endometrial cancers the majority of signature genes were up-regulated for eribulin treatment as compared to paclitaxel (76% and 56% in breast and endometrial cancer, respectively). On the other hand, in ovarian cancer the majority of signature genes were down-regulated for eribulin treatment compared to paclitaxel (74% of genes). Next we performed unsupervised clustering based on the gene sets in all three cancer panels for both treatments. We identified two distinct clusters based on eribulin expression profile and three clusters based on paclitaxel expression profiles in breast cancer (Figure 2). We found that the drug treated expression profiles for breast cancer correlated with sensitivity for both compounds (p = 0.004 for eribulin and p = 0.06 for paclitaxel; Table 1 and Figure 2). We considered the top two or three clusters in each case and reported the best p value. The choice of hierarchical clustering algorithm was determined based on the silhouette average, a measure which characterizes how tightly the data are grouped in the clusters (Material and Methods). For ovarian cancer we found no significant correlation of expression with drug sensitivity, and for endometrial cancer we found significant correlation only with the paclitaxel signature (p = 0.006, Table 1). Because breast cancer expression showed the most significant association with eribulin sensitivity, in the next part of the analysis we investigated in more detail the clustering results. We identified the most resistant clusters for eribulin (HCC1500, HCC1419, UACC893, HCC2218, UAC812) and paclitaxel (HCC1143, HCC2218, HCC1954, BT20, HCC70, UACC812), both characterized by distinct expression profiles (Figures 2A and 2B, indicated with red boxes) and significantly higher IC_50_ values from the rest of the cell lines (p = 0.004 and p = 0.06, respectively). The two resistant clusters shared two common cell lines (HCC2218 and UACC812) and had several unique ones. These cell lines were predicted based on the drug treated expression profile patterns to be uniquely resistant to one or the other compound. To compare the sensitivity of the cell lines to the two compounds we examined the scatter plot of the IC_50_ values of eribulin and paclitaxel. The scatter plot (Figure 2C) shows a weak correlation (correlation = 0.19, not significant) that means differences between antiproliferative activity between eribulin and paclitaxel in breast cancer panel, because there are several cell lines located off-diagonal in the top-left (most sensitive to eribulin as compared to paclitaxel) or bottom-right (most sensitive to paclitaxel as compared to eribulin) corner of the plot. These cell lines had the most difference in drug sensitivity between the two drugs. The clustering results predicted that HCC1500, HCC1419 and UACC893 are resistant to eribulin but not to paclitaxel. Indeed, two of the cell lines (Figure 2C, red) are the top two cell lines most resistant to eribulin compared to paclitaxel based on the scatter plot. We note that for the cell line UACC893 we could not determine the IC_50_ values because of slow cell growth issues in the in vitro proliferation assay. Similarly, three of four cell lines (HCC1954, HCC70, BT20) predicted by the clustering results are among the top most resistant to paclitaxel compared to eribulin (yellow) based on the IC_50_ scatter plot. The remaining cell line (HCC1143) appears to be resistant both to paclitaxel and eribulin. Although it was not part of the eribulin resistant cluster, the cell line shows similarity in gene expression profile (up-regulation) to the resistant cluster (Figure 2A). These results showed that in the breast cancer panel, drug resistant cell lines were characterized by distinct expression profiles which can differentiate eribulin and paclitaxel antiproliferative activity.

**Table 1:** Correlation of gene signatures with in vitro antiproliferative data.

Gene signatures	Breast cancer (p values)		Ovarian cancer (p values)		Endometrial cancer (p values)	
Eribulin signature	0.004		NS		NS	
Paclitaxel signature	0.06		NS		0.006	
**EMT pathway**	**Clustering**	**Prediction**	**Clustering**	**Prediction**	**Clustering**	**Prediction**
Eribulin (EMT)	0.06 (0.05)*	0.03	NS	NS	NS	0.04
Paclitaxel (EMT)	NS	NS	NS	NS	0.006	0.02

We performed unsupervised hierarchical clustering based on gene signatures for the 3 cancer panels. Significant (p<0.05) or marginally significant (p<0.1) p values are listed for the cell line panels where we identified clusters of cell lines with different sensitivities. For the EMT pathway we tested the predictive power of the expression profiles based on the elastic net regression model. The predicted and measured values (IC_50_) were correlated based on the Pearson correlation coefficient. In cases where significant correlations existed, p values are listed. Significance of EMT pathway clustering was confirmed for breast cancer by qPCR (p = 0.05). *confirmed by TLDA (NS indicates not significant p values>0.1).

### Functional analysis of gene signatures

The gene signatures in the cancer panels were quite unique; nevertheless, there was a small overlap of 18 genes. Interestingly a large fraction of the overlapping genes were tubulins (11 out of 18). These tubulins were down-regulated under eribulin treatment as compared to paclitaxel in most cell lines across all 3 cancer types. The significant differences in tubulin expression levels were confirmed by qPCR in the breast cancer panel. Based on the qPCR analysis, 4 tubulins (TUBA1B, TUBA4A, TUBB, TUBB4B) had significantly decreased expression under eribulin treatment compared to control (based on paired t-test, Table 2). In contrast, 7 of 8 tubulins (TUBA1B, TUBA1C, TUBA4A, TUBB2A, TUBB3, TUBB4B, TUBB6) had significant increases in expression under paclitaxel treatment compared to control. There was a large variation in fold-changes for the individual cell lines, but all 4 tubulins with significant changes under eribulin treatment had >1.5 fold changed decreases in expression in at least 11 cell lines in breast cancer. Similarly, 6 of 7 tubulins with significant changes had increases in expression of >1.5 fold in at least 7 cell lines under paclitaxel treatment when compared to control. All the tubulins tested by qPCR were significantly down-regulated under eribulin treatment when compared to paclitaxel (p<0.001 for all tubulins except TUBB6, p<0.05, Table 2). We found that reduced expression of 4 tubulins (TUBA1C, TUBA4A, TUBB3, TUBB6) significantly correlated with eribulin sensitivity (p<0.05), and that of one tubulin had a weak correlation (TUBB, p<0.1). Only one tubulin (TUBB2A) had significant correlation with paclitaxel sensitivity (p<0.05) and several (TUBA4A and TUBA1B) showed marginal significances (p<0.1, Table 2).

**Table 2:** Correlation of tubulin expression with drug sensitivity and fold changes between drug treatments.

	Eribulin		Paclitaxel		Eribulin vs control	Paclitaxel vs control	Paclitaxel vs Eribulin
Tubulin	pv	cor	pv	cor	Fold change	Fold change	Fold change
TUBA1B	NS	0.16	0.07	−0.38	0.75 (p<0.001)	1.17 (p<0.05)	1.87 (p<0.001)
TUBA1C	0.01	0.49	NS	−0.16	NS	1.31 (p<0.01)	1.61 (p<0.001)
TUBA4A	0.007	0.52	0.08	−0.36	0.84 (p<0.01)	1.64 (p<0.001)	2.42 (p<0.001)
TUBB	0.08	0.36	NS	−0.23	0.7 (p<0.001)	NS	1.65 (p<0.001)
TUBB2A	NS	−0.01	0.04	−0.44	NS	1.76 (p<0.001)	1.81 (p<0.001)
TUBB3	0.04	0.42	NS	−0.17	NS	1.68 (p<0.001)	1.67 (p<0.001)
TUBB4B	NS	0.06	NS	−0.2	0.78 (p<0.001)	1.39 (p<0.001)	2.1 (p<0.001)
TUBB6	0.02	0.48	NS	−0.03	NS	1.34 (p<0.01)	1.42 (p<0.05)

Correlations were calculated based on the Pearson correlation, and p values between treatment and controls are based on paired t-test (NS indicates a not significant p value>0.1).

The correlative analysis of the signature gene sets with altered expression between treatments indicated a significant correlation with drug sensitivity for breast and endometrial but not for ovarian cancer (Table 1). We investigated the functional role of the genes differentiating eribulin from paclitaxel based on gene expression by performing pathway enrichment analysis using the commercially available Metacore software. The three genes sets were used for enrichment analysis and a significance threshold of p<0.05 was applied with the FDR adjustment. Table 3 shows the significantly enriched pathways grouped as common and unique for the different cancer panels. There were several common pathways for the 3 cancer types: cytoskeleton remodeling related pathways (neurofilaments and keratin filaments), and the role of Nek in the cell cycle regulation pathway. Breast and endometrial cancers were uniquely enriched in two EMT related pathways (“Dependence of TGF beta independent induction of EMT via RhoA, PI3K and ILK” and “Development regulation of epithelial-to-mesenchymal transition”) and a cell adhesion pathway related to cell migration (“Cell adhesion chemokines and adhesion”). There was only one pathway uniquely enriched for endometrial and ovarian cancer (“Cell adhesion gap junction”). There were several unique pathways enriched for each cancer panel: 5 unique pathways significantly enriched for both breast and ovarian cancer and two unique pathways for endometrial cancer (Table 3). Most unique pathways were related to specific processes in cytoskeleton remodeling, cell cycle and immune response (Table 3). Additional pathways included regulation of eNOS pathway enriched for breast cancer, transcriptional role of AP1 for endometrial cancer, activin A signal regulation, and development of beta-adrenergic receptor transactivation of EGFR enriched for ovarian cancer.

**Table 3:** Pathway enrichment analysis of the three gene signatures altered between eribulin and paclitaxel treatments.

Breast cancer	Endometrial cancer	Ovarian cancer
Cytoskeleton remodeling (3.5×10^−7^,1.6×10^−3^, 9.3×10^−4^)		
Cell cycle: Role of Nek in cell cycle regulation (1.5×10^−4^, 4.7×10^−4^, 5.9×10^−8^)		
Cytoskeleton remodeling: Neurofilaments (1.3×10^−4^,1.6×10^−5^,1.2×10^−6^)		
Cytoskeleton remodeling: Keratin filaments (7.5×10^−4^, 1×10^−4^, 1.8×10^−4^)		
Development of TGF beta dependent induction of EMT via RhoA, PI3K and ILK (6.7×10^−6^, 2.2×10^−3^)		
Development regulation of epithelial-to-mesenchymal transition (EMT) (2.5×10^−5^,1.3×10^−3^)		
Cell adhesion Chemokines and adhesion (4.1×10^−5^, 1.4×10^−3^)		
	Cell adhesion gap junction (1.5×10^−4^, 5.8×10^−4^)	
Cytoskeleton remodeling_Integrinoutside-in signaling (1.5×10^−5^)	Immune response_IL-1signaling pathway (2.6×10^−4^)	Immune response_Human NKG2D signaling (1.4×10^−3^)
Cytoskeleton remodeling_TGF, WNT andcytoskeletal remodeling (3.8×10^−5^)	Transcription_Role of AP-1 inregulation of cellularmetabolism (1.5×10^−3^)	Immune response_Murine NKG2 signaling (1.5×10^−3^)
Cell cycle_Spindle assembly and chromosome separation (6.4×10^−5^)		Cytoskeleton remodeling_Reverse signaling by ephrin B (1.7×10^−3^)
Muscle contraction_Regulation of eNOS activity in endothelial cells (1.1×10^−4^)		Development_Beta-adrenergic receptors transactivation of EGFR (2×10^−3^)
Immune response_ETV3 on CSF1-promoted macrophage differentiation (3×10^−4^)		Signal transduction_Activin A signaling regulation (2.2×10^−3^)

**Figure 3:**
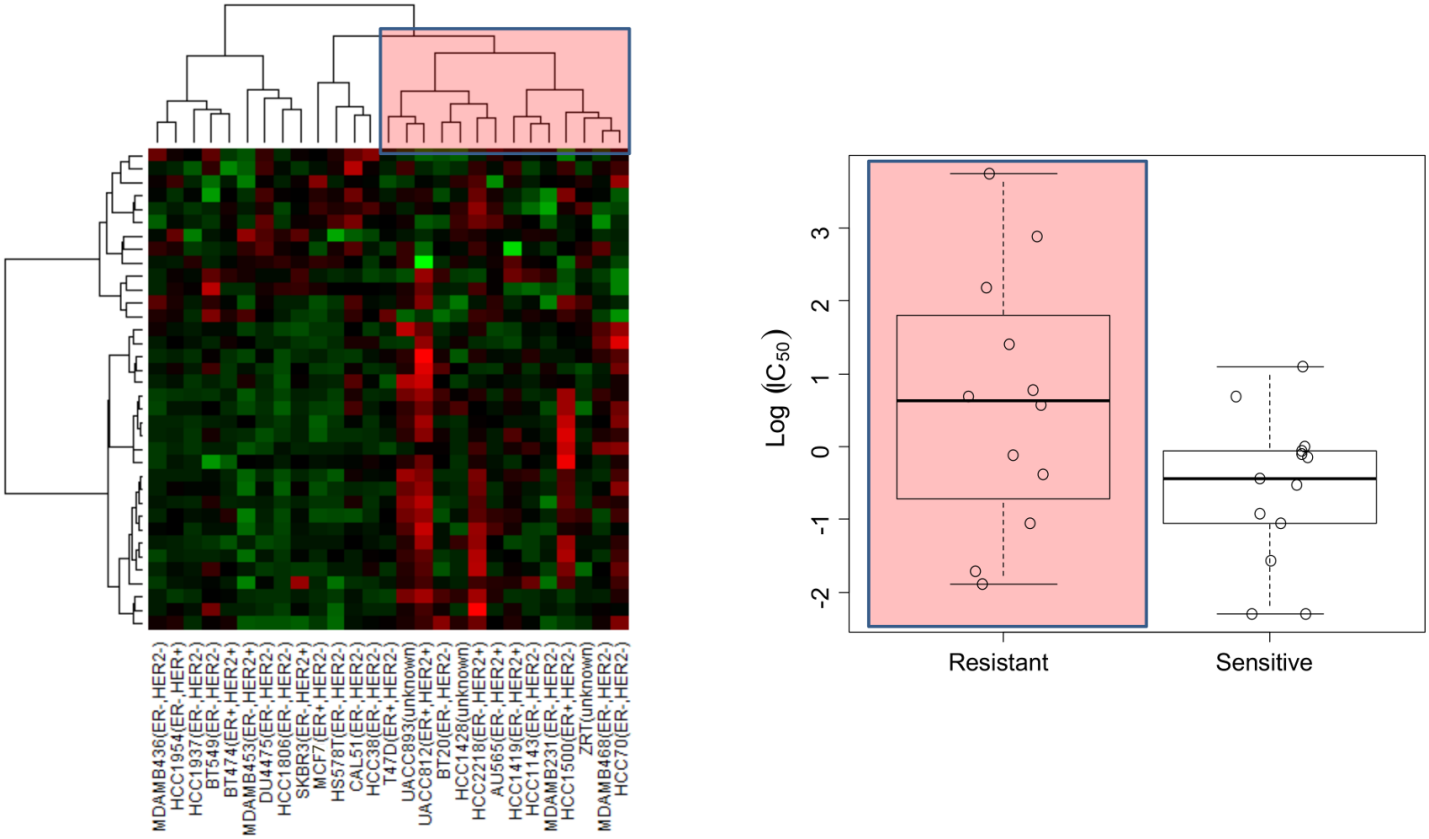
The EMT expression profile correlates with eribulin sensitivity. Unsupervised hierarchical clustering defined groups of breast cancer cell lines with altered expression under eribulin treatment (left panel). The cell lines consisting of many upregulated EMT genes are more resistant to eribulin treatment (right panel, p = 0.06). The ER and HER2 status of cell lines are indicated in the parenthesis.

The analysis revealed that two EMT related pathways were commonly enriched for breast and endometrial cancer. Interestingly these were the two cancer panels which had gene signatures with significant correlation with eribulin and/or paclitaxel sensitivity. We tested the hypothesis that EMT pathway being common in these two cancer types may be a selective pathway associated with drug sensitivity. To investigate the expression profile in more detail we extracted the list of genes from the “Development regulation of epithelial-to-mesenchymal transition” pathway. Out of the 91 genes 36, 33, and 41 genes were expressed in breast, ovarian, and endometrial cancer, respectively. First, we applied hierarchical clustering based on these genes for breast cancer and analyzed the sensitivity between two groups which were divided by hierarchical clustering. EMT pathway expression correlated with eribulin (p = 0.06; Table 1, Figure 3), but not with paclitaxel sensitivity (Table 1). For ovarian cancer there was no correlation between EMT gene expression and drug sensitivity. Lastly, EMT pathway expression had a significant correlation with paclitaxel sensitivity in endometrial cancer (p = 0.006). Next we tested if the differentiation of EMT gene expression profile between resistant and sensitive cell lines would enable us to predict drug sensitivity. The predictive power of the EMT expression profile was evaluated by applying the elastic net approach [18] with leave-one-out cross validation (See Materials and Methods). The predicted and measured drug sensitivity (IC_50_ value) had significant correlation in breast cancer (p = 0.03) and in endometrial cancer (p = 0.04) with eribulin sensitivity. The predicted drug sensitivity was also significant for paclitaxel in endometrial cancer (p = 0.02). On the other hand, there was no significant prediction power for ovarian cancer based on EMT expression profile. The predictions are in agreement with the unsupervised clustering results, suggesting that the separation observed in the EMT expression profile between sensitive and resistant cell lines in breast and endometrial cancer can be used to predict drug sensitivity. The agreement between these two different computational approaches also suggests the robustness of these findings. Although we have not found significant separation between eribulin sensitive and resistant cell lines based on the clustering of EMT expression profile in endometrial cancer, the genes from EMT pathway still had significant predictive power of eribulin sensitivity. This may be due to the fact that in this case the best predictive model in elastic net includes a relatively small number of genes from the EMT pathway as opposed to the clustering which is based on all genes expressed in EMT (7 genes from EMT are included in the model to predict eribulin sensitivity in endometrial cancer compared to 13 and 21 genes included in the models to predict paclitaxel sensitivity in endometrial cancer and eribulin sensitivity in breast cancer, respectively).

**Figure 4:**
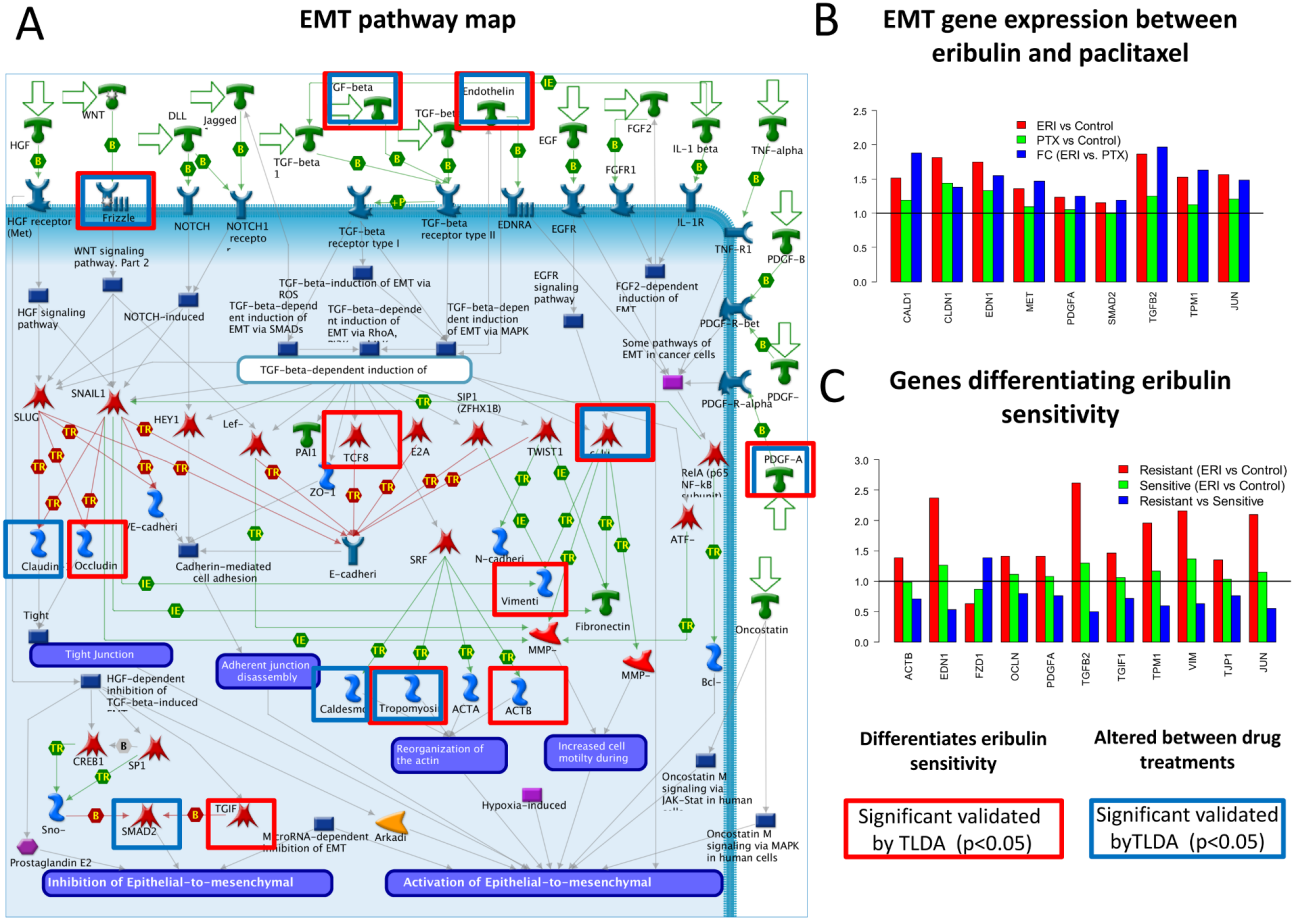
Gene expression profiles of EMT pathway. A) The EMT pathway. The boxes show genes with significantly different expression between eribulin sensitive and resistant cell lines (red) and with significantly different expression between eribulin and paclitaxel treatments (blue). B) EMT gene expression between eribulin and paclitaxel. The plot shows the fold-changes of significantly altered genes between paclitaxel and eribulin (red: eribulin vs. control; green: paclitaxel vs. control; blue: eribulin vs. paclitaxel). C) Genes differentiating eribulin sensitivity. The plot shows the fold-changes of significantly altered genes between eribulin sensitive and resistant cell lines (red: resistant; green: sensitive; blue: resistant vs. sensitive).

The EMT genes expressed in breast cancer (36 genes) were included in a custom TLDA assay to confirm our results. Based on the higher sensitivity TLDA assay, both EMT correlation with eribulin sensitivity (p = 0.05) and the significant alteration of EMT expression between eribulin and paclitaxel sensitivity was confirmed. We found that 9 genes from the EMT pathway had significantly higher expression (p<0.05) under eribulin treatment when compared to paclitaxel, supporting our results from the microarray data (Figure 4B). These 9 genes had a large variation across the cell line panels, but they were mostly up-regulated and the fold-changes were larger than 1.5 for at least 3 cell lines. Eleven genes had significantly higher expression under eribulin treatment for the resistant cluster when compared to the sensitive cell line cluster (Figure 4C). A detailed investigation of the EMT pathway map revealed that several genes identified as overexpressed under eribulin treatment or overexpressed in the eribulin resistant cell lines were key elements of the epithelial to mesenchymal process (Figure 4A). EMT is mediated by a number of signaling pathways, including TGF-beta, WNT, EGF, and HGF signaling [19]–[22]. Our results indicated a significant alteration of expression in key EMT marker genes such as vimentin and claudin-1 (upregulated under eribulin treatment compared to paclitaxel) and occludin (upregulated in eribulin resistant cell lines). Indeed, overexpression of claudin-1 was shown to induce EMT through activation of slug and zeb1 in human liver cells [23], and to mediate TNF-alpha induced cell migration in human lung carcinoma [24]. Another important gene of the EMT pathway is TGF-beta, which is able to induce the EMT pathway via the SMAD family members [22]. Our map indicates over-expression of both TGF-beta and SMAD2 suggesting a possible mechanism for the activation of the pathway. We found that endothelin-1 was significantly overexpressed under eribulin treatment compared to paclitaxel as well as in the eribulin resistant cell lines. The TGF-beta 1 dependent secretion of endothelin-1 is known to be associated with the induction of the EMT [25]. These findings based on gene expression profiling suggest that EMT pathway response differentiates eribulin from paclitaxel and it is uniquely associated with eribulin sensitivity in breast cancer cell lines.

## Discussion

The similarity of the signatures of altered expression profiles for the 3 cancer panels was analyzed based both on gene and pathway levels. Although the gene level overlap was very small (only 18 genes) among the 3 panels, the pathway analysis revealed that several common or functionally closely related pathways were enriched (related mostly to cell cycle, cytoskeleton remodeling, and immune response). Our analysis showed that the tubulins were significantly downregulated under eribulin treatment compared to paclitaxel. The downregulation under eribulin treatment and the upregulation under paclitaxel treatments of several tubulins was validated by qPCR in breast cancer (Table 2). The alteration of expression profiles of cell cycle, cytoskeleton remodeling and immune response pathways under paclitaxel treatment has been reported previously both in vivo and in vitro in ovarian cancer [26], [27], which was consistent with our data including breast and endometrial cancer panel. The upregulation of tubulin expression and changes in expression of cytoskeleton maintenance genes under paclitaxel treatment has also been reported in rat smooth muscle cells [28]. Previous studies indicated that alpha- and beta-tubulin synthesis is auto-regulated by posttranscriptional mechanisms that can alter mRNA levels of tubulins in response in the unassembled tubulin subunit concentration [29]. Based on these findings, the upregulation of tubulin expression by paclitaxel is likely driven by a compensatory mechanism to increase the supplies of tubulin monomers depleted because of the microtubule polymerization, whereas the downregulation of tubulins by eribulin could be explained by the opposite effect of increasing the number of tubulin monomers by inhibiting microtubule polymerization [30]. Although expression profiles of tubulins treated with paclitaxel has been analyzed in vitro before, a comprehensive analysis of tubulin alteration under eribulin treatment and comparison with paclitaxel has not yet been done. Furthermore, our correlation analysis revealed unique sets of tubulins correlating with eribulin sensitivity which could potentially allow us to identify subpopulations with relative benefit to eribulin compared to paclitaxel. These findings need further investigation and validation.

The analysis of expression profiles revealed that the EMT pathway was significantly altered between the two treatments. EMT is a process in which epithelial cells acquire mesenchymal properties and show loss of intercellular cohesion, increased cellular migration, and increased resistance to apoptosis and anticancer agents. In tumor cells, EMT may increase the motility and invasiveness of cancer cells and is thought to play a fundamental role during invasion and metastasis (for example [31], [32]). In previous studies, the EMT signature has been found to correlate in vitro with cisplatin resistance in ovarian cancer [33], [34], and resistance to gemcitabine, 5-FU and cisplatin in pancreatic cancer [35]. Moreover, EMT regulators have been shown to modulate resistance to EGFR inhibitors in bladder cancer [36]. Our findings indicate that in breast cancer, EMT pathway expression correlates with eribulin resistance, but not with paclitaxel resistance. Detailed analysis of the EMT pathway after mapping significantly altered genes based on qPCR revealed that many key elements of the signaling pathway were significantly altered both between eribulin sensitive and resistant cell lines and between treatments with the two compounds. Vimentin, one of the important markers of EMT pathway activation, was significantly upregulated in eribulin resistant cell lines. Several studies have reported overexpression of vimentin as a marker of basal-like breast cancer cells [37], [38]. Furthermore, it has been shown that overexpression of vimentin in the non-invasive MCF7 breast cancer cell line increases invasiveness [39].

In this study, it is not clear if eribulin will cause EMT in resistant cancer cells, because we determined limited gene signature in the EMT pathway a short time after treatment (24 hour). Eribulin caused MET, which is an opposite to EMT, in one of triple negative breast cancer cell line, which has a mesenchymal phenotype [40]. Additionally, we examined the EMT gene expression 7 days after eribulin treatment for 5 selected breast cancer cell lines (Table S4). Thirteen genes out of 48 from EMT pathway showed consistent down-regulation (Table S5) in three cell lines sensitive to eribulin based on EMT expression profiling (Figure 3). This indicates that eribulin may cause MET change in vitro as well in the sensitive cell lines. These pre-clinical findings additionally suggest that based on specific alterations in EMT gene expression we may identify the sub-population of patients with more benefit from eribulin treatment compared to paclitaxel treatment. These patients may be identified by rapidly analyzing the gene signature of EMT pathway after the drug treatment.

Our correlation analysis indicated an interesting interrelatedness of the EMT pathways association with eribulin and paclitaxel sensitivity among the 3 cancer panels. We found that alterations in EMT expression are predictive of eribulin sensitivity in breast cancer and with both eribulin and paclitaxel sensitivity in endometrial cancer. We found no significant correlation in ovarian cancer. These in vitro findings may be related to the similarity of the molecular profiles among some cell lines from the 3 cancer panel. Recently the development of several high content assays provided an opportunity for the comprehensive analysis of the molecular profiles of the breast, endometrial, and ovarian cancers [41]–[43]. Integrated analysis of mRNA expression, DNA copy number, DNA methylation and sequencing data revealed a similarity among the molecular profiles in sub-types of these cancers. The molecular profiling revealed a common molecular signature among basal-like breast cancer, serous ovarian and serous endometrial cancers. This profile was markedly different from the other endometrial and luminal breast cancer types. The mRNA clustering of endometrial cancer defined 3 robust clusters of subtypes called the “mitotic”, “hormonal” and “immunoreactive”. The hormonal endometrial cancer showed similar molecular profile to the luminal breast cancer which is hormone receptor positive. The finding that both breast and endometrial cancer are related to the sensitivity of the drugs may be related to the fact that the similar molecular profile is driving the sensitivity to treatment and not the cancer type. For example the observation that the breast and endometrial EMT gene signature was predictive of eribulin sensitivity may be related to subtypes of breast and endometrial cancer with common molecular profiles. Indeed we found the highest similarity between endometrial and breast cancer based both on gene and pathway level analysis. Breast cancer signatures shared twice as many genes with endometrial cancer compared to ovarian cancer when the overlapping genes were normalized to the relative sizes of the gene sets. The majority of the genes were upregulated for eribulin treatment compared to paclitaxel for the breast cancer (76%) and endometrial cancer (56%) signatures as opposed to the ovarian cancer signature, where the majority of genes were down regulated (74%). The finding that the EMT signature correlated with paclitaxel sensitivity only for endometrial cancer may be indicative of a cancer subtype with a unique molecular profile. Alternatively, the endometrial cancer subtype resistant to paclitaxel may have similar molecular profiles to some breast or ovarian cancer subtypes not included in the cell line panels.

In summary, the correlative analyses and results of predictions based on the EMT signature in breast and endometrial cancers suggest that EMT pathway genes may serve as biomarkers to predict response to eribulin in breast cancer, and eribulin and paclitaxel in endometrial cancer. Additionally, our findings indicate that the gene signature can separate eribulin resistant subgroups from paclitaxel resistant subgroups in breast cancer panel. We found that tubulin isotopes had significantly lower expression in cell lines treated with eribulin compared to paclitaxel. Lastly, we found that reduced expression of 4 tubulins (TUBA1C, TUBA4A, TUBB3, TUBB6) significantly correlated with eribulin sensitivity and that of one tubulin (TUBB2A) significantly correlated with paclitaxel sensitivity.

## Supporting Information

Table S1 (XLSX)

Table S2 (XLSX)

Table S3 (XLSX)

Table S4 (XLSX)

Table S5 (XLSX)
